# Older People at Risk of Suicide: A Local Study During the COVID-19 Confinement Period

**DOI:** 10.3390/healthcare13141735

**Published:** 2025-07-18

**Authors:** Ismael Puig-Amores, Guadalupe Martín-Mora-Parra, Isabel Cuadrado-Gordillo, Jessica Morales-Sanhueza

**Affiliations:** 1 Department of Psychology and Anthropology, University of Extremadura, 06071 Badajoz, Spain; ipuigamores@unex.es (I.P.-A.); cuadrado@unex.es (I.C.-G.); 2 Directorate of Humanistic and Christian Formation, General Directorate of Teaching, Catholic University of Temuco, Rudecindo Ortega, Temuco 2950, Chile; prof.moralessanhueza@gmail.com

**Keywords:** elderly people, suicide, COVID-19, mental health

## Abstract

Background: Suicide in older adults represents an insufficiently addressed public health problem, despite the aging population and the increase in mental disorders in this group. The COVID-19 pandemic and associated measures, such as lockdown, could have exacerbated this phenomenon. This study aimed to analyze the impact of the confinement decreed during the state of alarm in Spain on the incidence of deaths by suicide in people over 70 years of age in Extremadura. Methods: An observational and retrospective study was carried out, using data from the Institutes of Legal Medicine and Forensic Sciences, comparing the figures for 2020 with the years 2019, 2021, along with the average for the period 2015–2019. Statistical analyses included Chi-square tests and calculation of Relative Risk with 95% CI. Results: The results revealed a significant increase in deaths by suicide in the third quarter of 2020 compared to the periods compared, especially among men. Conclusions: It is concluded that confinement may have negatively influenced the mental health of older adults, which underscores the need for specific interventions and attention to regional contextual factors.

## 1. Introduction

Suicide in older people is a topic of increasing importance, especially in the context of an aging population and the additional challenges generated by the COVID-19 pandemic. Although not all older people experience suicidal thoughts, there is a prominent correlation between aging and an increased risk of suicide [1]. Despite the above, death by suicide (DBS) in older people has been studied less compared to younger groups, and the importance of this phenomenon in individuals over 60 years of age has been minimized [2,3]. However, the progressive aging of the population marks the severity of suicide in this age group [4]. In this sense, it is estimated that, by 2050, the world population of people over 60 will double, reaching 2100 million. In addition, the number of people over 80 years of age is expected to increase by three times between 2020 and 2050, reaching 426 million [5]. Likewise, it is also expected that depression will become the main cause of mortality and morbidity in the world [6], and it is expected that this problem will also be of great relevance among the elderly population. In this context, recent studies [4] point to the importance of specialized mental health care in the elderly, even more so considering the evidence indicating that older adults do not seek specialized mental health care. As a result, it is necessary to transfer research to primary care services and the community [2].

During the COVID-19 pandemic, seniors have been one of the most vulnerable populations, so much so that 90% of recorded COVID-19 deaths occurred in individuals over 60 years of age as well as three-fifths of people aged 80 years and older in 21 OECD (Organization for Economic Co-operation and Development) countries [7]. In addition, the disease in its acute phase has generated neuropsychological and psychological problems in the infected population [8], with a deterioration in executive functions, attention, and memory in post-COVID-19 patients [9,10]. The pandemic has also caused notable changes in lifestyle behaviors in the Spanish population, especially in relation to diet, sleep, and physical activity [11]. Social distancing measures and quarantines have generated emotional distress worldwide, negatively impacting mental health [12]. Efforts to contain the spread of the virus have contributed to increased social isolation in the elderly, generating a significant impact on their mental and physical health in this age group [13,14,15,16]. Consequently, during the initial phase of the epidemic, more than 50% of the general population reported significant psychological impact, with approximately one-third experiencing moderate-to-severe levels of anxiety [17]. Likewise, García-Álvarez et al. [18] revealed a considerable psychological impact in the first days of confinement, with significant differences between groups with and without a history of mental disorder. Overall, mental health disorders have increased substantially worldwide [19].

In the older age group, several factors have been associated with suicide attempts, including, but not limited to, a history of psychiatric treatment and previous suicide attempts [20,21]. Likewise, the suicide mortality rate increases with age, being higher in men in all age groups, especially in the group over 75 years of age [22].

During the pandemic, early studies initially indicated that global suicide numbers remained stable or decreased in the first few months [23]. However, subsequent studies have observed that overall suicide figures for certain sex- and age-based groups were higher than expected [24,25,26,27]. In particular, a significant increase in suicide deaths has been observed in the elderly group in some geographical areas [28,29,30,31].

In Spain, the results found are contradictory with respect to the rise in suicides for years. Thus, a study in the region of Catalonia revealed a 6.3% decrease in suicidal behavior rates recorded in 2020 compared to 2019, with no significant changes in overall suicide mortality rates [32]. However, De la Torre et al. [33] identified an increased risk as of May 2020, although no significant differences were observed in overall suicide mortality rates between 2019 and 2020. Other studies [34,35] showed lower-than-expected monthly suicide counts in April 2020, peaking in August 2020, with a noticeable, albeit not statistically significant, increase in global figures [35] or in men over 65 years of age [34].

The pandemic also had global repercussions on mental health services. According to a study by the World Health Organization (WHO) in the second quarter of 2020, more than 60% of countries reported interruptions in the provision of mental health services [36]. Thus, despite a higher prevalence of suicidal thoughts in individuals affected by COVID-19, until October 2020, there was a reduction in hospital visits related to suicidal behavior [37]. Thus, disparities in estimates of mental health disorders and treatment indicate a notable imbalance between the potential need for and use of mental health services during the pandemic [38]. In addition, there are signs of an increase in demand and unmet needs for mental health care in OECD nations [19]. In this regard, it is found that, during the first wave of COVID-19, those with moderate/severe symptoms of depression and anxiety, as well as with a worse mental health self-assessment, were more likely to go to health services. Paradoxically, advanced age (over 65 years of age) was associated with a reduction in the use of these services [39]. Likewise, the joint presence of a suicide attempt within that year and a major depressive episode were associated with consultation with a primary care physician and, to a greater extent, with the use of Mental Health services [40,41]. After the confinement period, an increase in consultations for autolytic attempt and ideation has also been observed [42,43,44]. Some findings suggest that certain demographics of older patients were at increased risk of depression and anxiety during COVID-19 [45].

In this context, the analysis of the events that occurred during the pandemic is considered of vital importance to understand the factors associated with the increase in suicidal ideation and suicide attempts in the elderly, a population that is especially vulnerable to COVID-19 and its consequences. Not paying attention to these consequences can lead to their maintenance in the short, medium, and long term, leading to the expected increase in deaths caused by suicide in the elderly population. Thus, the present research was carried out in the region of Extremadura (Spain), with a total census population of 1,063,565 inhabitants, of which 18% are people over 70 years of age. The annual rate of deaths by suicide in this region has maintained a remarkable stability in the last five years. A previous study determined the absence of any seasonal pattern in suicide cases during the years 2015–2019 in this region [35]. Likewise, no significant difference was found globally in deaths by suicide in 2020 compared to the previous 5 years. In this regard, however, it should be noted that the lack of information and opacity in relation to the topic made it very difficult to analyze the phenomenon of suicide in older people in the aforementioned study [35]. Therefore, the performance of new analyses focused on cases of suicide during the period of confinement and post-confinement can complete and expand the previous results, consequently providing relevant data for the detection and prevention of the phenomenon. With this, the objective of this work was to determine to what extent and direction the quarantine period decreed during the state of alarm in Spain could have been associated with a change in the recorded incidence of deaths by suicide in people over 70 years of age in Extremadura. Consequently, the following hypothesis was formulated:

During the quarantine period in 2020, there was a significant increase in suicide deaths among people over the age of 70 in the region of Extremadura (Spain).

## 2. Materials and Methods

### 2.1. Study Design

The study carried out is observational and retrospective. The analysis compared deaths by suicide in 2020 with previous and subsequent years, as well as with the period of confinement. A year-on-year comparison was made to analyze the variation in the incidence of DBS observed in the four quarters of 2020 versus the incidence in the same periods of the previous and subsequent years (2019 and 2021) as well as with the average of the previous 5 years (2015–2019), considering for exposed and non-exposed groups the total population and by age groups registered in Extremadura for each year (under 40 years; between 40 and 69 years old; over 70 years of age). The COVID-19 lockdown period was defined as from March to June 2020, and the post-lockdown period as from July to October 2020.

For the analysis, the statistical software SPSS 26 (IBM Corp., Armonk, NY, USA)—a package that allowed comparisons to be made by calculating contingency tables and obtaining the values of Chi-Square (X^2^) and Relative Risk (RR) with their 95% confidence interval—was used. This method is justified by its ability to provide a simple estimate of the percentage variation between incidence rates and has been used in other studies to calculate the association between periods of confinement and cases of DBS, as well as the risk of suicide during the COVID-19 period [26,46,47,48,49,50,51,52]. 

The resulting RR value was used to determine the probability of DBS in the group exposed to confinement relative to the unexposed group. An RR value > 1 would indicate a significant increase in DBS when the lower bound of the CI is above 1, while an RR value < 1 would indicate a significant decrease when the upper limit of the CI is below 1.

### 2.2. Participants

The study population comprises all cases of suicide deaths registered in Extremadura (Spain) from 1 January 2015 to 31 December 2021 (N = 381; Men 81.6%; Women 18.4%). The age ranged from 16 to 94 years ($\overset{—}{x}$ = 54.83; SD = 18.78). The distribution by age group was 22% under 40 years of age, 52.3% between 40 and 69 years of age, and 25.7% of people aged 70 or older.

### 2.3. Data Collection and Procedure

The data used in this study were obtained from the suicide registries of the Institutes of Legal Medicine and Forensic Sciences of the region of Extremadura, with the approval of the Teaching Commission of these centers and the authorization of the Ministry of Justice of Spain. A systematic review was conducted on the violent death registry books, identifying unnatural causes between 2015 and 2021. Following an individual review of the forensic reports, only cases unequivocally classified as suicide were included. The data were anonymized and coded, in compliance with Organic Law 3/2018, of 5 December, on the Protection of Personal Data and guarantee of digital rights. 

## 3. Results

Starting with the comparison of deaths by suicide, the data show that, during the year before and after the start of the pandemic, a lower number of cases were recorded in people over 70 years of age, especially in the third quarter. As for the rest of the age groups, a notable decrease in deaths by suicide in people under 40 years of age can be observed during 2021 compared to 2020. There was also a decrease in the phenomenon in people between 40 and 69 years of age (X^2^ = 5.237; *p* = 0.022) (RR = 0.4; CI = 0.17–0.9) during the third quarter of 2020 compared to the previous year.

**Figure 1:**
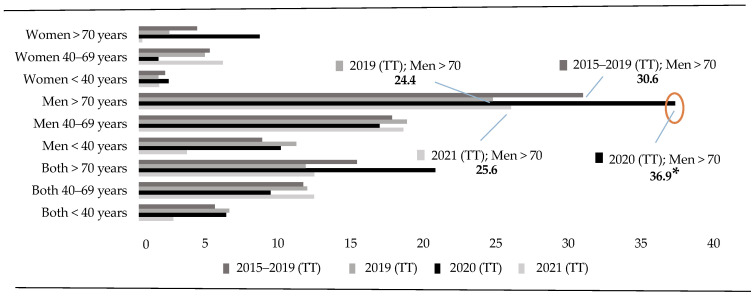
Annuals Rates (<40 vs. 40–69 vs. >70 years). Note: Total Trimester (TT) represents the four trimesters of the year. * *p* < 0.05.

In the analysis of the total number (i.e., considering both sexes) of deaths by suicide registered in 2020, a significant increase in DBS among people over 70 years of age can be observed in the third quarter of 2020 compared to the same period within the last 5 years (X^2^ = 7.311; *p* = 0.007) (RR = 3.28; CI = 1.32–8.16). This increase is also observed when compared to 2019 (X^2^ = 7.445; *p* = 0.006) (RR = 3.31; CI = 1.33–8.24) and 2021 (X^2^ = 12.470; *p* = 0.000) (RR = 6.63; CI = 1.97–22.31). This fact indicates that the DBS curve increased in 2020 to decrease again in the following year, at the beginning of the pandemic, before reaching the lowest rates recorded in the last 7 years (Figure 1).

**Figure 2:**
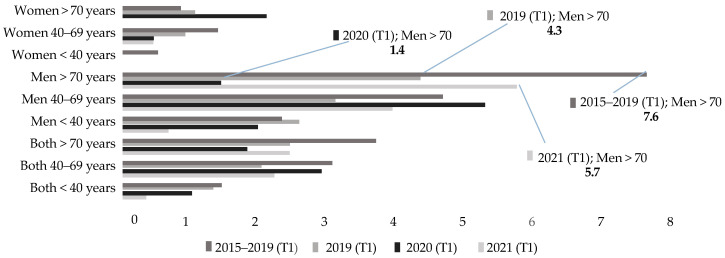
First trimester rates (T1) (<40 vs. 40–69 vs. >70 years).

Figure 2 shows the distributions of DBS rates recorded during the first trimester (T1) of the years from 2015 to 2021. As can be seen, in the last three years of the period analyzed (2019–2021), the rates recorded in people over 70 years of age are lower than the average of the last 5 years. Additionally, during the first months of the pandemic, for this age group, the lowest rate in the series was recorded (1.42). In more detail, Figure 2 shows how this figure decreases compared to previous years and then returns to initial values, with figures even higher than those in the records prior to the start of the health crisis. This result points to the increase in deaths by suicide among the elderly in the post-pandemic period. However, the statistical analyses in this quarter have not provided significant results for any age group. 

**Figure 3:**
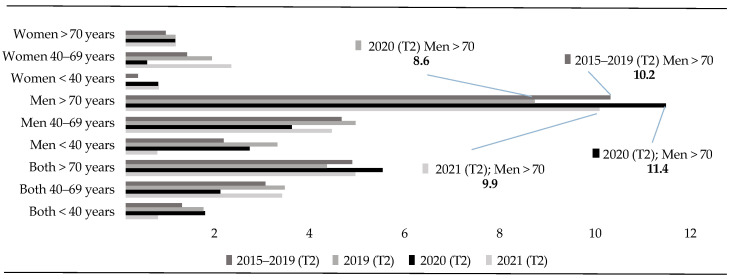
Second trimester rates (T2) (<40 vs. 40–69 vs. >70 years).

In the same vein, the analysis of the second quarter of the years between 2015 and 2021 (Figure 3) reveals some stability in the suicide death rates recorded in all age groups.

**Figure 4:**
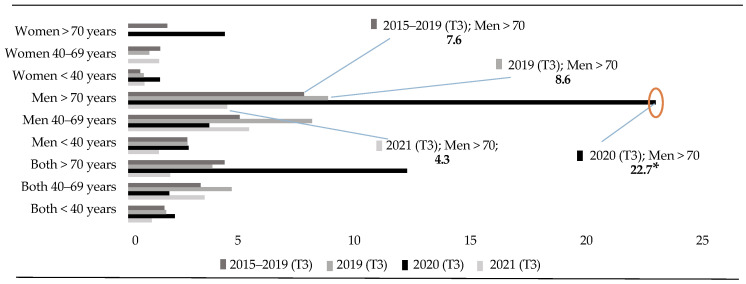
Third trimester rates (T3) (<40 vs. 40–69 vs. >70 years). Note: (*) *p* < 0.05.

On the other hand, Figure 4 shows that, in the third quarter (T3) of 2020, a period that coincides with the first months after confinement, almost 4 times more deaths of men over 70 years of age were recorded than in the same period within the last 5 years. Likewise, the data reveal an increase in deaths by suicide in this age group that is two and a half times more than in the same period of 2019, and more than five times higher than in the same period during the post-pandemic period (2021). This finding shows that, in the quarter after the confinement of 2020, there was an increase in DBS among men over 70 years of age, and then a sharp decrease in these was observed in the same period of the following year. This decrease in deaths by suicide reaches an even lower rate than in the 5 years prior to the pandemic.

Continuing with the analysis, and focusing attention on the third quarter of 2021, a significant decrease in the total number of deaths registered is observed. This decrease corresponds to a lower number of cases registered among people over 70 years of age. Additionally, during 2020, there was a decrease of up to three times in deaths of men under 40 years of age (Figure 4).

**Figure 5:**
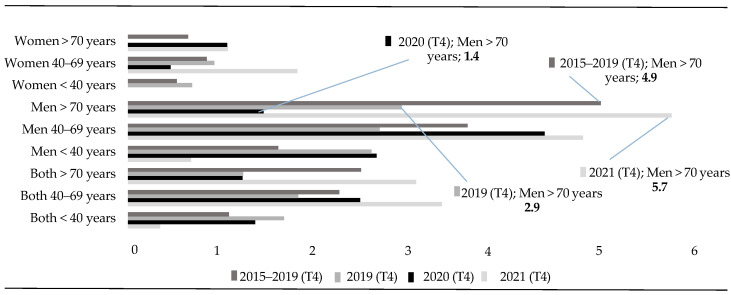
Fourth trimester rates (T4) (<40 vs. 40–69 vs. >70 years).

Regarding suicide mortality rates during the fourth quarter (T4) of 2020, a notable difference is observed in the rate for the group of men over 70 years of age when compared to the rest of the years (Figure 5). In this period of 2020, the suicide mortality rate decreased significantly to 1.4 (RR = 0.06; CI = 0.01–0.35), a figure much lower than that recorded in the same months of the previous five years ($\overset{—}{x}$ = 4.9; RR = 0.25; CI = 0.07–0.93) and in 2021 (5.7; RR = 3.97; CI = 1.55–10.16).

In summary, in 2020, a significant increase in DBS has been observed in people over 70 years of age when compared to 2019 (X^2^ = 7.445; *p* < 0.01) and 2021 (X^2^ = 12.470; *p* < 0.0 001). However, this increase is not found when the comparison is made with the average of the 5 years prior to the pandemic. This increase responds to an unusual record of DBS of people over 70 years of age during the three months following the confinement period. In this quarter (Q3), statistically significant differences are also observed when the data compare suicide deaths recorded in adults over 70 years of age (regardless of sex) in the previous five years (*p* < 0.01). Likewise, in this third quarter, there was a significant increase in deaths by suicide in the group of men over 70 years of age compared to 2019 (X^2^ = 4.451; *p* = 0.035) (RR = 2.6; CI = 1.03–6.7), 2021 (X^2^ = 7.553; *p* = 0.003) (RR = 5.3; CI = 1.5–18.3) and the mean of the 5 years prior to the pandemic (X^2^ = 6.904; *p* = 0.009) (RR = 3.9; CI = 1.3–11.7). In contrast, there is no evidence in either direction in the group of women in this age group during the third trimester.

Finally, the analysis in the rest of the age groups analyzed (between 40 and 70 years old), only in 2021 was a notable decrease in DBS observed in the group of people under 40 years of age. This data indicates that people in this age group had up to a two and a half times (CI = 1.06–6.07) smaller risk of suicide than in the year when the pandemic began (2020) (X^2^ = 4.679; *p* = 0.031). Specifically, this lower risk was observed in the group of men of this age (RR = 0.338; CI = 0.1–0.930) (X^2^ = 4.857; *p* = 0.028) (Table 1 and Table 2).

## 4. Discussion

The main objective of this study was to evaluate the impact of the confinement period during the state of alarm on the incidence of suicides among people over 70 years of age in the region of Extremadura. In this regard, the increase in suicidal ideation during confinement and post-confinement, as well as its relationship with the COVID-19 pandemic, has been the subject of several studies. Gelezelyte et al. [53] reported an increase in suicidal ideation during this period. Likewise, a meta-analysis by Dubé et al. [54] highlighted the high prevalence of suicidal ideation related to COVID-19 symptoms, post-pandemic behavioral changes, and mental health factors. On the other hand, an increase in suicidal behavior (ideation and attempts) has been observed in young people globally [55,56], and, in the case of military veterans, those infected with COVID-19 were at significantly higher risk of suicidal ideation [57]. In addition, a review by Sinyor et al. [58] found positive associations between SARS-CoV-2 and COVID-19 infection and subsequent suicidal/self-injurious thoughts, both in the general population and in health care workers. These findings underscore the importance of addressing mental health in the context of the pandemic and its repercussions on suicidal ideation and behavior.

In this global context, our research is part of the studies that examine suicide trends during the pandemic [24]. In this regard, the findings of the present study are consistent with those suggesting that the COVID-19 pandemic could have had a significant impact on suicide rates, particularly among people over 70 years of age. Indeed, our results have been replicated to a greater or lesser extent in some geographical areas of the world, such as Japan [26,59], France [60], Italy [61], and Germany [47,49,50,51,52]. 

In the same vein, our data point in the same direction as those revealed in Spain by De la Torre-Luque et al. [33] and Martínez-Alés et al. [34], whose monthly suicide counts showed an upward trend in deaths by suicide after the period of confinement in men over 65 years of age and revealed that there was no increase in the number of suicides during the period of confinement (from March to June 2020) compared to previous years. This increase in the number of suicides during the three months after the quarantine period was also observed when comparing the data with the same three-month period in 2019 and 2021. This finding is consistent with the stability of the prevalence of suicide in men for years. Thus, while women are more likely to perpetrate suicide attempts, men have shown a higher percentage of deaths by suicide [62], a phenomenon that is consistently found in periods of previous economic crises such as the one that occurred in 2008 [47,63,64]. 

The explanation for the gender differences is not easy to find. Thus, although various works have provided varied and heterogeneous interpretations (choice of more lethal means by men, cultural and social differences in the expression of suffering, etc.) [62,65,66,67], none of them seems to be complete [68], especially in periods of crisis such as the one analyzed in this paper. In this regard, they suggest that the effects of psychiatric disorders have a greater marginal effect on men than on women, a fact that could also explain the higher number of suicides in periods of maximum stress [68]. It is also possible that the extraordinary circumstances caused by the pandemic may have contributed to this increase in suicide rates. For example, unexpected deaths, as well as restrictive measures taken with respect to the celebration of funeral rites, family and social gatherings, etc., with the aim of preventing the spread of the COVID-19 virus, could have become a source of stress and complication of grief for these elderly men, who, in addition, tend to show more problems expressing feelings in the face of losses [69]. 

The next revealing fact from the analysis of results is that the group of people over 70 years of age are the ones who committed the most suicidal acts in the post-confinement period, which is striking when compared to previous studies. Thus, the results of the present study also allow us to observe that the worsening in the data could have been transitory. In fact, the analysis reveals that during the year before and after the start of the pandemic (i.e., 2019 and 2021), fewer cases were recorded in people over 70 years of age, especially in the third quarter, suggesting that the effects of the pandemic on suicide rates may have been temporary.

The explanation for these events may be related to the extraordinary events that occurred during the period of confinement. The health crisis caused by COVID-19 led to an undoubted increase in people’s isolation [53], a factor that could have been even greater among older people who, to a large extent, already showed high levels of loneliness [70,71]. Likewise, increased feelings of anger, fear, and bewilderment, increased stress, as well as an increased risk of mental illness and cognitive impairment have been pointed out [71,72]. It is also important to consider the difficulties in accessing primary care and social services that revealed their operational weaknesses during this period [73]. The combination of these factors could have been responsible for the increased risk of suicide [74,75,76] in the immediate aftermath of the mandatory lockdown, especially in Spain, which had one of the highest incidences in Europe [77]. 

Additionally, comparing the data of this age group with younger groups of subjects, the results reveal a notable decrease in the number of deaths by suicide in people under 40 years of age during 2021 (post-confinement period), compared to 2020 (confinement period). Likewise, there is a decrease in the number of deaths by suicides between 40 and 69 years of age during the third quarter of 2020 compared to the previous year. Thus, despite the increase in the phenomenon observed in 2020, the suicide rate decreased in the year following the start of the pandemic, reaching the lowest rates recorded in the last 7 years [41]. Consideration of the extraordinary circumstances experienced during the pandemic can help, once again, to understand this finding. In this way, the return to normality after the mandatory confinement period decreed by the government could become a protective factor for the health of middle-aged adults. In this regard, research that has analyzed previous crisis situations (e.g., economic crises) has pointed out that it is middle-aged men who lose their jobs who are most likely to commit suicide [63]. Likewise, the economic and social measures taken by the Government of Spain that protected the work of many citizens (Economic and Social Measures in the COVID-19 Crisis, 2020) could be the key to reducing the phenomenon in this age group. In addition, the repercussions of the quarantine would have been fewer in this age group, which presented less mortality and risk, and that could, to a certain extent, regain contact with their social support network outside the family. These events could mark an improvement in the mental health of this group of adults.

## 5. Limitations

Research on mortality rates in the health field faces several limitations that affect the interpretation of the results and the generalizability of the findings. The quality of medical records and death certificates can vary, resulting in underreporting or inaccurate information. Causes of death can be misclassified, either due to human error or limitations in coding criteria. The complex interactions between risk factors may not be fully understood or controlled, so more detailed research focusing on these factors would be necessary to detect protective and risk elements for the mental health care of the elderly and suicide prevention. In this sense, the study must acknowledge the limitation that potential confounding factors (e.g., pre-existing mental health conditions, socioeconomic status, or access to health care) were not taken into account due to data availability constraints. The limited availability of detailed data makes it difficult to identify specific patterns or risk groups. Finally, Spain is characterized by a wide cultural diversity, as well as marked differences in sociodemographic and socioeconomic characteristics across its various regions. In this context, it is important to consider that Extremadura has its own specific features in these areas, which means that the result obtained from studies conducted in this geographical area may not be directly generalizable to other regions of the country with different contexts.

## 6. Conclusions

The COVID-19 pandemic has highlighted the importance of protecting older people and generated reflections on how to best address their needs in the future. The importance of post-COVID-19 psychological support has been pointed out [78], a finding that underscores the importance of access to mental health services to mitigate the negative impact of crises. The World Health Organization (WHO) has highlighted the need to collect high-quality data on the effects of the pandemic on the mental health of the population and vulnerable groups, as well as on the brain function, cognition, and mental health of COVID-19 patients. In this regard, our specific approach to analysis in the region of Extremadura and in the population over 70 years of age provides a detailed analysis of temporal variations, free of seasonality and autocorrelation in the data, as supported by previous studies [35]. These elements underscore the importance of considering regional and demographic particularities when interpreting the consequences of events such as the pandemic on the incidence of suicides. It is also relevant to point out that our study is based on data collected directly from the Institute of Legal Medicine and Forensic Sciences (IMLyCF) of Extremadura, which provides a greater guarantee of veracity and accuracy of the data compared to the National Institute of Statistics, where compromising underreporting has been documented for any statistical treatment of deaths by suicide [79,80,81].

Consequently, the present study has important practical applications. Firstly, taking into account, on the one hand, that suicide attempts are more frequent among people who suffer or have suffered from psychological and emotional disorders [82,83,84], and, on the other hand, that a significant proportion of completed suicides are associated with mental health problems [85], and given that this and other cited studies show that suicide attempts can increase after a period of health alert [86], the urgency of investigating how to mitigate the consequences on mental health under pandemic conditions is emphasized. Likewise, it is necessary to analyze the impact of the implementation of extraordinary measures established by health agencies and the government related to crisis situations, as well as the differential effect that these measures have on the gender differences found in suicide rates. Especially in older adults, it would be necessary to consider the predisposing factors characteristics of old age (e.g., chronic illnesses or dependency), precipitating factors (such as loneliness and quality of life), and clinical indicators in mental health [87].

Second, it requires the development, evaluation, and improvement of interventions designed to address the psychological, social, and neuroscientific aspects of the pandemic [78]. Mental health services play a critical role in providing a safe environment for people to address their concerns and develop strategies to manage stress [35,41]. Mental health professionals can also offer practical tools and emotional support to help people cope with economic challenges and build resilience. Therefore, it is crucial to advocate for the normalization of the use of mental health services, as well as to educate the community about the importance of mental health care, especially in difficult times.

Finally, the need to implement strategies that guarantee access to health care and address the social and emotional implications of the pandemic on the lives of older people is underlined. Given the increasing trend and the projected growth of the population aged over 70 in the region of Extremadura [88,89], these strategies should include measures to reduce social isolation, ensure access to adequate health services, and foster social connection and emotional well-being through community-based programs. Likewise, awareness, education, and eliminating stigma around mental health in old age are crucial steps to prevent suicide and improve the quality of life for older people. The susceptibility of older people to serious diseases and the complications arising from these diseases has led to greater attention to their needs and the implementation of specific measures to protect them. In addition, it has been observed that the perception of social support improves explicit memory and quality of life and reduces depression in active older adults [90]. With this, it is of vital importance to consider the previous mental state when addressing the psychological effects of the pandemic [41], which, together with the aforementioned recommendations, could strengthen, to a certain extent, the prevention of the ideation and consummation of the suicidal act.

For future research, it is recommended to broaden the geographical scope by including data from other regions of Spain, in other to improve the representativeness and generalizability of the findings. It would also be advisable to user larger samples that allow for more robust analyses. Furthermore, future studies should aim to more precisely examine the casual impact of restrictions, taking into account potential intervening or confounding variables that may influence the results. To this end, in order to help clarify the underlying causal relationship, research with a more theoretical approach is needed, such as that based on the most current explanatory models of suicidal behavior (Interpersonal Theory of Suicide; the Integrated Motivational-volitional Model of Suicidal Behavior; the Three-Step Theory) [91,92,93]. 

## Figures and Tables

**Table 1:** Suicide cases by Year and Gender.

			Total TT			Trimester 2			Trimester 3	
	Year	<40 Years Old	40–69 Years Old	>70 Years Old	<40 Years Old	40–69 Years Old	>70 Years Old	<40 Years Old	40–69 Years Old	>70 Years Old
Men	2015–2019	14.0	39.2	21.0	3.4	10.2	7.0	4.2	10.8	5.2
	2019	17	42	17	5	11	6	4	18	6
	2020	15	38	26	4	8	8	4	8	16
	2021	5 *	42	18	1	10	7	2	12	3
Women	2015–2019	2.8	10.6 *	3.8	0,4	2.8	0.8	0.8	3.0	1.6
	2019	2	10 *	2 *	0	4	1	1	2	0
	2020	3	3	8	1	1	1	2	0	4
	2021	2	13 *	2 *	1	5	1	1	3	0
Both	2015–2019	16.8	50.0	24.6	3.8	13	7.8	5	13.8	6.8
	2019	19	52	19 *	5	15	7	5	20	6
	2020	18	41	34 *	5	9	9	6	8	20
	2021	7 *	55	20 *	2	15	8	3	15	3

NOTE: Year-on-year analysis of 2020 versus 2015–2019 and the immediate preceding and following years. Rate Ratio (RR); 95% CI; * *p* < 0.05.

**Table 2:** Comparison of the Relative Risk of suicide in 2020 compared to other years.

			Total TT			Trimester 2			Trimester 3	
	Year	<40 Years Old	40–69 Years Old	>70 Years Old	<40 Years Old	40–69 Years Old	>70 Years Old	<40 Years Old	40–69 Years Old	>70 Years Old
Men	2015–2019	-	-	-	-	-	-	-		3 (1.12–8.07) *
	2019	-	-	-	-	-	-	-	0.44 (0.19–1.02)	2.64 (1.03–6.75) *
	2020	-	-	-	-	-	-	-	-	-
	2021	2.96 (1.07–8.14) *		-	-	-	-	-	-	5.32 (1.55–18.26) *
Women	2015–2019	-	0.27 (0.08–0.97) *	–	–	–	–	–	–	–
	2019	–	0.3 (0.08–1.08)	3.98 (1.33–11.91) *	-	-	-	-	-	-
	2020	-	-	-	-	-	-	-	-	-
	2021	-	0.23 (0.07–0.82) *	3.97 (1.33–11.87) *	-	-	-	-	-	-
Both	2015–2019	-	-	-	-	-	-	-	-	2.89 (1.21–6.91) *
	2019	–	–	1.77 (1.01––3.11) *	–	–	–	–	0.4 (0.17–0.9) *	3.31 (1.33–8.24) *
	2020	-	-	-	-	-	-	-	-	-
	2021	2.53 (1.06–6.07) *	-	1.69 (0.97–2.94)	2.96 (1.07–8.14) *	-	-	-	-	6.63 (1.97–22.31) *

NOTE: Year-on-year analysis of 2020 versus 2015–2019 and the immediate preceding and following years. Rate Ratio (RR); 95% CI; * *p* < 0.05.

## Data Availability

The original contributions presented in this study are included in the article.
